# Improving the recovery and detection of bloodstream pathogens from blood culture

**DOI:** 10.1099/jmm.0.001209

**Published:** 2020-06-03

**Authors:** Kerry Falconer, Robert Hammond, Stephen H. Gillespie

**Affiliations:** ^1^​ School of Medicine, University of St Andrews, St Andrews, UK

**Keywords:** bloodstream infection, blood culture and bacterial recovery

## Abstract

**Introduction.:**

Bloodstream infections (BSI) are growing in incidence and present a serious health threat. Most patients wait up to 48 h before microbiological cultures can confirm a diagnosis. Low numbers of circulating bacteria in patients with BSI mean we need to develop new methods and optimize current methods to facilitate efficient recovery of bacteria from the bloodstream. This will allow detection of positive blood cultures in a more clinically useful timeframe. Many bacterial blood recovery methods are available and usually include a combination of techniques such as centrifugation, filtration, serum separation or lysis treatment. Here, we evaluate nine different bacteria recovery methods performed directly from blood culture.

**Aim.:**

We sought to identify a bacterial recovery method that would allow for a cost-effective and efficient recovery of common BSI pathogens directly from blood culture.

**Methods.:**

Simulated *E. coli* ATCC 25922 blood culture was used as a model system to evaluate nine different bacteria recovery methods. Each method was assessed on recovery yield, cost, hands-on time, risk of contamination and ease of use. The highest scoring recovery method was further evaluated using simulated blood cultures spiked with seven of the most frequently occurring bloodstream pathogens. The recovery yield was calculated based on c.f.u. count before and after each recovery method. Independent *t*-tests were performed to determine if the recovery methods evaluated were significantly different based on c.f.u. ml^−1^ log recovery.

**Results.:**

All nine methods evaluated successfully recovered *E. coli* ATCC 25922 from simulated blood cultures although the bacterial yield differed significantly. The MALDI-TOF intact cell method offered the poorest recovery with a mean loss of 2.94±0.37 log c.f.u. ml^−1^. In contrast, a method developed by Bio-Rad achieved the greatest bacterial yield with a mean bacteria loss of 0.27±0.013 log c.f.u. ml^−1^. Overall, a low-speed serum-separation method was demonstrated to be the most efficient method in terms of time, cost and recovery efficiency and successfully recovered seven of the most frequent BSI pathogens with a mean bacteria loss of 0.717±0.18 log c.f.u. ml^−1^.

**Conclusion.:**

The efficiency of bacterial recovery can vary significantly between different methods and thereby can have a critical impact on downstream analysis. The low-speed serum-separation method offered a simple and effective means of recovering common BSI pathogens from blood culture and will be further investigated for use in the rapid detection of bacteraemia and susceptibility testing in clinical practice.

## Introduction

Bacteria can invade and multiply in the normally sterile bloodstream and this can have devastating clinical outcomes with bloodstream infections (BSI) associated with high mortality (20–46 %) and morbidity [1]. BSI caused by Gram-negative bacteria is of great concern with high rates of antimicrobial resistance [2–4]. *E. coli* is the most frequently isolated Gram-negative bacteria in BSI and is responsible for over 20 % of cases worldwide [5].

Blood culture is the gold-standard for the diagnosis of BSI but is hindered by the long incubation time required to detect the very low circulating levels of bacteria (1–100 c.f.u. ml^−1^) [6–9]. After the detection of BSI an overnight culture is required for the identification (ID) of the causative pathogen and associated antimicrobial susceptibility (AST). Overall, this results in a delay in providing evidence-based treatment decisions by up to 48–72 h [10].

To optimize the workflow from patient to pathogen ID and AST, a variety of recovery methods have been developed for isolating bacteria directly from blood. Some of these recovery methods include mechanical filtration, centrifugation, sedimentation, red blood cell lysis, chemical capture on surfaces or beads and microfluidic techniques [11–18]. Recovering bloodstream pathogens efficiently from the patient is vitally important in order to confidently rule in or rule out bacterial infection, to identify the causative agent and perform AST accurately and quickly. A method that enables bacterial ID and AST directly from positive blood culture would offer a significant reduction in turn-around time by removing the need for overnight culture.

In this study we evaluated a range of currently available blood-culture techniques for recovering *E. coli* directly from blood culture and examined the efficiency offered by each method. Other frequent BSI pathogens were used to further examine the influence of bacteria *spp*. on recovery efficiency.

## Methods

### Bacteriological methods

*Escherichia coli* ATCC 25922 was used as a model organism to initially evaluate nine different recovery methods. The highest scoring recovery method was further evaluated by cultivating an additional six BSI pathogens, these were *Staphylococcus aureus* ATCC 29213, *Klebsiella pneumonia *e ATCC 700603, *Pseudomonas aeruginosa ATCC* 27853*, Acinetobacter baumannii ATCC* 19606*, Streptococcus agalactiae ATCC* 12386 and *Streptococcus pneumoniae ATCC* 49619. ATCC reference strains were donated by Victoria Hospital, Kirkcaldy, UK and identified by Vitek 2. All strains were stored in glycerol stock at −80 °C until required. Cultures were grown from glycerol stock, by inoculating a 10 µl loopful of stock into 10 ml Brain-Heart Infusion (BHI) broth (Sigma Aldrich, UK). Cultures were incubated overnight at 37 °C in aerobic conditions (static incubation with lose cap) and each culture was grown to an optical density (OD_600_) of 0.6.

### Preparation of surrogate blood culture

Blood culture refers to a blood sample inoculated into a rich broth media such as tryptic soy broth (TSB) to support optimal bacterial growth. A blood-to-broth ratio of between 1 : 5 to 1 : 15 is required to remove the antibacterial effects of human blood [19–21]. A 1 : 10 blood-broth ratio was used in this study and this was consistent for all methods evaluated. Defibrinated horse blood (HB035, TCS Biosciences), TSB (Sigma Aldrich, UK) and a spiked concentration range of 10^2^ to 10^6^ c.f.u. ml^−1^ *E. coli* ATCC 25922 was used to create a series of mock blood cultures to assess all nine recovery methods. Simulated blood cultures spiked with a bacterial concentration of 10^5^ c.f.u. ml^−1^ were used to study recovery yield of all other BSI pathogens. The starting bacterial concentration in blood culture and the bacterial recovery yield was determined by c.f.u. using BHI plate counts based on the Miles and Misra method [22]. Colonies were counted at the highest dilution when visible in all three replicates after overnight incubation at 37 °C. Averages were taken and c.f.u. ml^−1^ were calculated.

### Bacteriological recovery methods

#### Intact cell method

The intact cell method (ICM) for bacterial recovery was originally described by Ferreira and colleagues for performing direct bacterial identification from blood culture by matrix-assisted laser desorption ionization time-of-flight mass spectrometry (MALDI-TOF) [23]. Here, an adapted version of ICM was performed. A total of 4 ml of simulated blood culture was centrifuged at 2000 ***g*** for 30 s, the supernatant was centrifuged at 15 500 ***g*** for 5 min and the bacterial pellet was washed with 1 ml of deionized water for 15 s. The sample was centrifuged again at 15 000 ***g*** for 5 min and was re-suspended in 1 ml BHI broth. Aliquots were taken for dilution plates to assess bacterial recovery by change in c.f.u. ml^−1^.

#### Serum separation tube centrifugation

A protocol published by Barnini and colleagues was assessed [13]. An 8 ml portion of simulated blood culture was transferred into an 8.5 ml serum separator tube (SST) (Advanced SST Becton Dickson, UK). The sample was centrifuged at 2000 ***g*** for 10 min and aliquots of the supernatant were used to perform a dilution plate to assess bacterial recovery. The protocol was repeated again, however the centrifugation step was changed to 1500 ***g*** for 10 min to assess the impact of centrifugation speed on bacterial recovery.

#### Lysis-filtration

Two in-house methods adapted from Fothergill and colleagues were developed to assess a combination of lysis and filtration in the recovery of bacteria directly from blood culture [14]. Protocol A involved treating a 9 ml simulated blood culture with 1 ml of 0.1 % Saponin (Alfa Aesar, UK) for 5 min. The resultant lysate was then filtered through 5 µM filter (Millipore, EMD, UK) and into a fresh 15 ml falcon tube. Protocol B involved osmotic lysis by diluting the simulated blood culture 1 : 100 in sterile deionized water. The resultant lysate was then passed through a 5 µM filter. Aliquots of the resultant lysate were taken to assess recovery yield by dilution plate (c.f.u. ml^−1^).

#### Lysis-centrifugation

A method reported by Lupetti and colleagues was evaluated [24]. A 7 ml portion of a simulated blood culture was treated with 0.01 % Saponin for 15 min at room temperature. The sample was then transferred to a SST and underwent centrifugation at either 1500 ***g*** or 2000 ***g*** for 10 min. Aliquots of the supernatant were serially diluted and used to perform a series of dilution plates to determine bacterial recovery yield (c.f.u. ml^−1^).

#### Bio-Rad β LACTA test protocols A and B

Both methods were performed in accordance with the manufacturer’s instructions (Bio-Rad, Marnes-la-Coquette, France) [25]. For protocol A, 2 ml of simulated blood culture was transferred into an 8.5 ml SST and centrifuged at 2000 ***g*** for 10 min. The supernatant was removed and the sediment of bacteria present on the gel was washed in 1 ml sterile water. The sample was centrifuged at 300 ***g*** for 3 min and 800 µl of the sample was transferred into a microcentrifuge tube. Centrifugation at 15 000 ***g*** for 1 min allowed a bacterial pellet to be obtained and this was re-suspended in 1 ml of BHI media. For protocol B, an aliquot of 1 ml of blood culture underwent centrifugation at 1000 ***g*** for 1 min. The supernatant was discarded and the remaining pellet was treated with Triton X-100 solution at 0.1 % and mixed for 10 s by vortexing. A second centrifugation of 13 000 ***g*** for 1 min allowed a bacterial pellet to be obtained. The bacteria were re-suspended and washed with 1 ml of sterile deionized water. Centrifugation at 13 000 ***g*** for 1 min allowed the bacterial pellet to be re-suspended in 1 ml of BHI media. Aliquots of bacterial re-suspensions were used to perform a dilution plate and to calculate bacterial recovery yield (c.f.u. ml^−1^).

### Data analysis

Bacteria recovery yield was the calculated log difference between the expected and observed c.f.u. plate count, each method was performed in triplicate and is expressed as mean±sem. Independent *t*-tests were used to determine if a statistically significant difference was present between different recovery methods and recovery yield. *P*<0.05 was considered significant.

All data analysis was performed using GraphPad Prism 7.04.

## Results

### Evaluation of *E. coli* ATCC 25922 recovery yield by method comparison

**Fig. 1.**
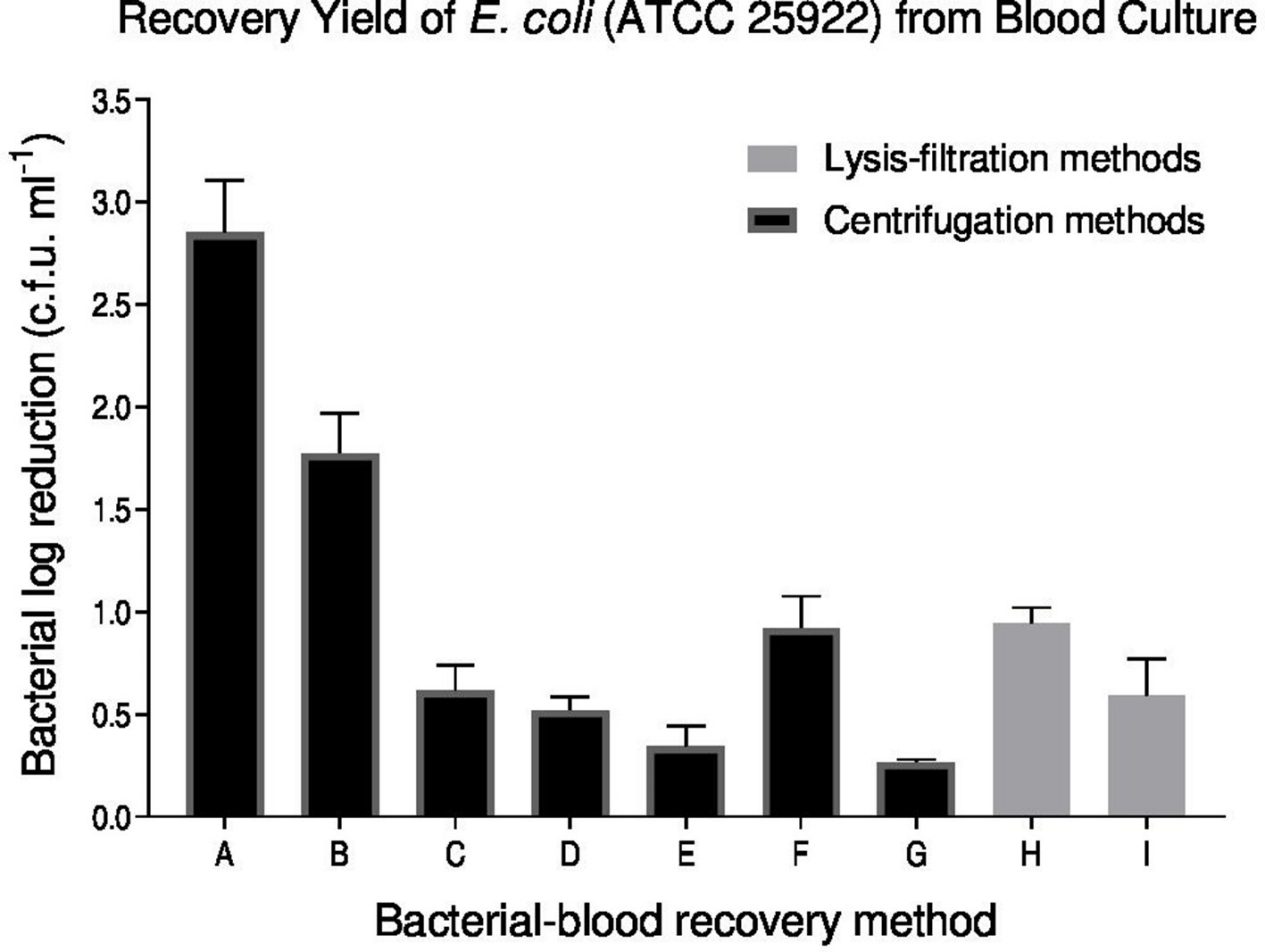
Comparison of bacteria yield from blood culture. Centrifugation-based methods included (a) the intact cell method (3.45±0.22 to 2.04±0.05 log c.f.u. ml^−1^), (b and c) SST centrifugation at two different centrifugation speeds (2000 ***g*** 1.14±0.56 to 2.29±0.54 log c.f.u ml^−1^ and 1500 ***g*** 0.29±0.02 to 0.91±0.14 log c.f.u. ml^−1^), (d and e) SST centrifugation with saponin (2000 ***g*** 0.32±0.45 to 0.60±0.40 log c.f.u. ml^−1^ and 1500 ***g*** 0.07±0.27 to 0.54±0.33 log c.f.u. ml^−1^),(f and g) Bio-Rad PR A and PR B (PRA 0.65±0.10 to 1.36±0.17 log c.f.u.ml^−1^ and PR B 0.22±0.11 to 0.27±0.089 log c.f.u. ml^−1^). Filtration-based methods for bacterial recovery from blood culture included (h) Lysis by osmosis and 5 µM filtration (0.41±0.67 to 1.08±0.07 log c.f.u. ml^−1^) and (i) lysis by 0.01 % saponin and 5 µM filtration (0.73±0.03 to 1.12±0.32 log c.f.u. ml^−1^).

Comparison of all nine methods showed each method to recover *E. coli* successfully (Fig. 1). However, the recovery yield obtained across the different methods was significantly different ranging from a log c.f.u. ml^−1^ loss of 0.26 to 2.94 bacteria (*P*<0.005). The intact cell method had the poorest bacterial yield from blood culture and was significantly different from all other methods tested (*P*<0.01). In contrast, Bio-Rad protocol B was observed to have the greatest recovery yield with only a small change between the spiked and recovered bacterial concentration (see Fig. 1g). Other methods such as serum separation by SST centrifugation was optimal at the lower centrifugation speed of 1500 ***g*** compared to 2000 ***g*** offering good recovery yields of less than one log c.f.u. ml^−1^ loss (*P*=0.001). The addition of a lysis pre-treatment with 0.1 % saponin before SST centrifugation at 2000 ***g*** significantly enhanced recovery to less than 0.5 log c.f.u. ml^−1^ loss (*P*=0.001). However, pre-treatment with 0.1 % saponin did not significantly enhance recovery at 1500 ***g*** SST centrifugation. Alternatively, the two lysis-filtration-based methods tested were not significantly different with a bacteria reduction of 0.4–1 log c.f.u. ml^−1^.

In addition to evaluating the bacterial recovery yield offered by each recovery method it was also equally important to consider the impact on cost-effectiveness and the time involved in performing each method. Table 1 summarizes all of these aspects for all nine methods.

**Table 1.** Evaluation of nine different recovery methods direct from blood culture

Recovery method	Recovery rate (mean c.f.u. log change*)	Hands-on time/total time	Cost per sample	Ref.
Serum separation (2000 ***g***)	1.14–2.29	5 min/15 min	£0.37	[13]
Serum separation (1500 ***g***)	0.29–0.91	5 min/15 min	£0.37	[13]
Bio-Rad recovery protocol A	0.65–1.36	25 min/30 min	£0.42	[25]
Bio-Rad recovery protocol B	0.22–0.27	25 min/30 min	£0.19	[25]
Serum separation+chemical lysis (2000 *g*)	0.32–0.60	5 min/20 min	£0.38	[24]
Serum separation+chemical lysis (1500 *g*)	0.07–0.54	5 min/20 min	£0.38	[24]
Chemical lysis and filtration	0.73–1.12	20 min/25 min	£2.86	[14]
Natural lysis and filtration	0.44–1.08	10 min/15 min	£2.86	[14]
MALDI-TOF (intact cell method)	2.04–3.45	25 min/30 min	£0.069	[23]

Table 1 shows the recovery yield varied significantly with each method ranging from a log loss of 0.07 to 3.45 bacteria. The hands-on time and turnaround-time per method ranged from 5 to 25 min and 15–30 min, respectively. All methods evaluated were inexpensive ranging from £0.069–£2.86. *The mean log c.f.u. change was calculated over a concentration gradient of 10^2^ to 10^6^ c.f.u. ml^−1^.

### The recovery yield of seven common BSI pathogens using low speed serum separation

**Fig. 2.**
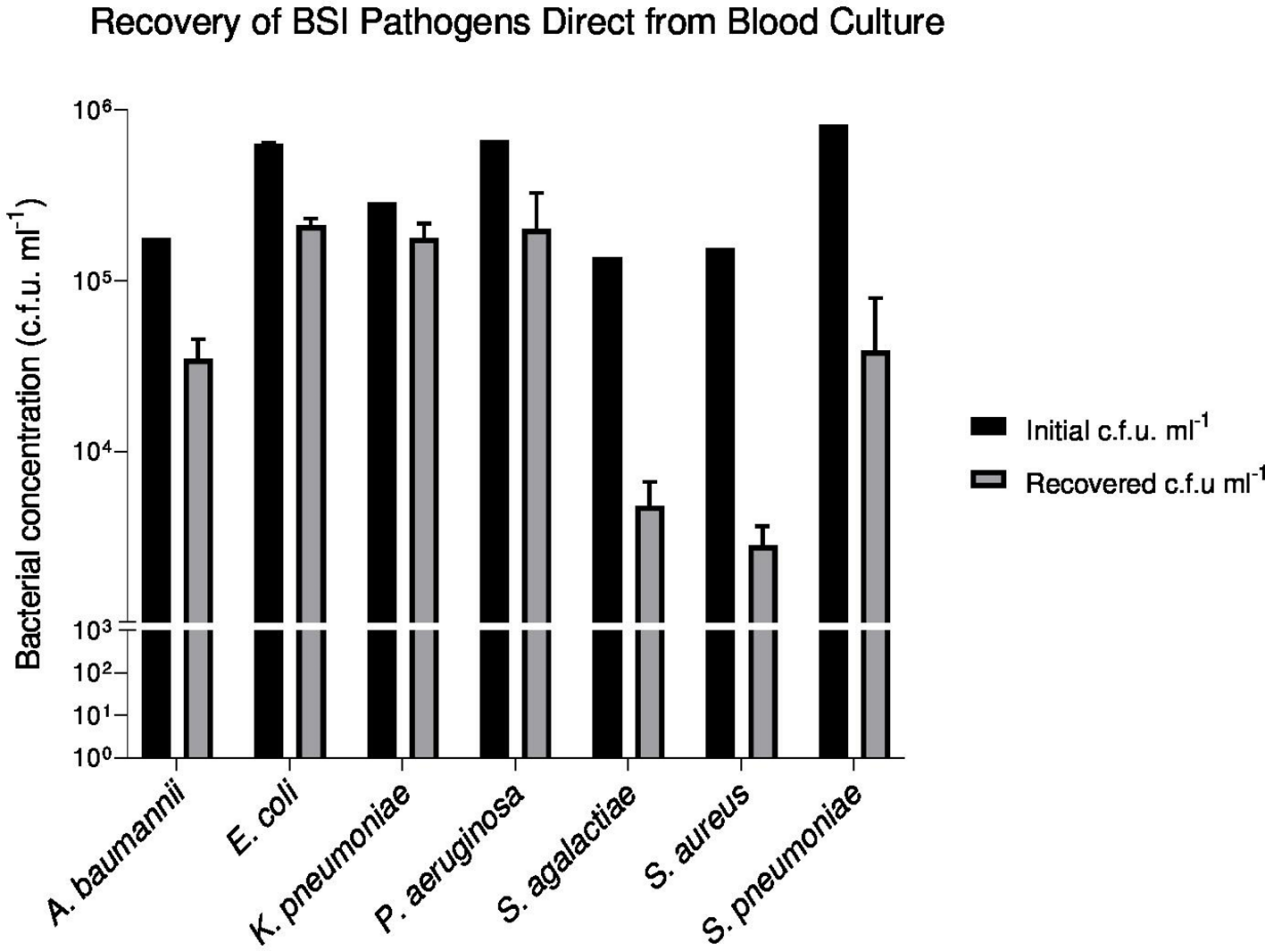
Recovery comparison of the most frequently associated BSI pathogens using SST centrifugation (1500 ***g***). The LS-SST method successfully recovered 7/7 BSI pathogens from simulated blood culture spiked at 10^5^ c.f.u. ml^−1^. The mean bacterial log reduction across BSI pathogens was 0.717±0.18 log c.f.u. ml^−1^ (*P*<0.05).

The low-speed serum-separation (LS-SST) method achieved the best combination of good recovery yield with low cost and quick preparation time. The study, therefore, evaluated the LS-SST method for the direct recovery of the most common BSI pathogens from simulated blood culture. Fig. 2 demonstrates that the LS-SST method facilitated the successful recovery of all seven BSI pathogens from blood culture. However, the recovery yield of bacteria was statistically significantly different among BSI pathogens and ranged from 0.21 to 1.74 c.f.u. ml^−1^ log loss (*P*<0.05). *K. pneumonia *e and *E. coli* presented with the highest recovery rates with *S. aureus* presenting with the lowest recovery of all the pathogens tested.

## Discussion

Early detection of bacteraemia has been shown to significantly improve patient care [1]. One way of improving the time to detection is to perform analysis directly from blood cultures, which potentially could save up to 24 h [26, 27]. This is one of few studies that has investigated and quantified the bacterial loss associated with extracting bacteria directly from positive blood cultures [28–33]. Recovery efficiency was evaluated for each of the nine methods by quantifying the recovery yield by colony plate counts (c.f.u. ml^−1^). *E. coli* was selected as a model organism to initially screen for the best recovery method – a predominant BSI pathogen accountable for up to 20–30 % of BSI cases [5, 34]. Here, we focussed on methods that would offer a rapid, cost-effective and transferable solution for extracting bacteria from blood culture in a low-cost and high throughput setting.

Ideally, a simple protocol with minimal steps is required in order to process blood cultures quickly; minimize the risk of contamination and reduce bacterial loss due to transfer. The hands-on time for the methods included in the study ranged from 5 to 25 min and the number of steps involved ranged from 3 to 10. A protocol from Bio-Rad provided the highest efficiency. The Bio-Rad protocol involves seven steps in total including three centrifugation steps, one lysis treatment and one washing step, with a total process time of 30 min. The scalability and time required to perform this method may make it less favourable in a high-throughput environment. Nevertheless, the most advantageous aspect of this approach is the high bacterial yield obtained. The ability to support the recovery of the very low bacteria counts commonly isolated from bacteraemia patients will enable improvement in time to detection where the starting concentration of blood culture is directly linked to time to positive ID and detection of antimicrobial resistance [35]. This is of high importance especially in low-volume blood samples or when a slow-growing pathogen is suspected of causing bacteraemia, both of which require a long incubation time and can take up to 5 days to become detectable on current blood-culture detection platforms [36].

Filtered-based methods have been shown to offer good bacterial recovery rates in this study and in previous studies [11]. Nonetheless, filtration-based methods may not be applicable for processing a large number of samples with greater technicality and more time required per sample, which in turn heightens the risk of contamination. Another consideration is the blood sample must be diluted to avoid a blood-filter cake forming, which may present an issue for low concentration and low-volume samples, for example in paediatrics [37]. Additionally, filtered-based methods were around nine times more expensive for each sample compared to centrifugation-based methods evaluated. The opportunity for automation using filtered-based methods is currently limited, which may present a challenge in streamlining this method into the workflow of some laboratories.

Most promising was the adapted SST method that offered a high recovery yield in a two-step process and further investigation demonstrated the LS-SST method to provide high recovery rates for seven common BSI pathogens with as little as 5 min hands-on time. SST offers a simple way to remove the high concentrate of haemocytes from the sample while leaving the bacteria intact in the serum. The removal of haemocytes from the sample is imperative for bacterial growth with the antibacterial effects of blood long been recognized [38]. Additionally, the purity of the bacteria extract will influence the accuracy and detection of molecular and phenotypic assays [39]. Barnini and colleagues have importantly demonstrated a SST method to facilitate rapid AST direct from blood culture in 6 h and due to the simplicity of approach and low-cost, this method can be applied and used to support many other direct and rapid bacteria ID and AST technologies [13].

*E. coli* was used as a model organism to screen numerous methods, followed by a full evaluation of the overall best method against the most common BSI pathogens. These include *A. baumannii, K. pneumoniae, P. aeruginosa, S. agalactiae, S. aureus* and *S. pneumoniae,* which are collectively responsible for 60.5 % of BSI cases worldwide [5]. This allowed the effective study of numerous recovery methods and the variation of recovery efficiencies amongst bacteria *spp.* Notably, *S. aureus* and other Gram-positive bacteria had lower recovery efficiencies in comparison to Gram-negative bacteria. Determining recovery efficiencies by c.f.u. ml^−1^ may have underestimated the true size of the active bacterial population in the case of Gram-positive bacteria. Gram-positive bacteria tend to aggregate and grow in grape-like clusters (cell-masses) or typical arrangements (chains) compared to Gram-negative bacteria that tend to grow as single rods [40]. This difference in growth characteristics and typical bacterial arrangement between Gram-positive and Gram-negative bacteria may also be attributed to method-specific differences. For example, the clustering and surface adherence of *S. aureus* may have reduced the ability to recover these bacteria when using the selected SST method. This highlights that one recovery method for all BSI pathogens may not be the optimal approach. Instead further screening using a Gram-positive organism such as *S. aureus* may favour an alternative recovery method and may be valuable in recovering Gram-positive bacteria directly from blood. However, the high bacterial load of blood culture, normally in excess of 10^8^ c.f.u. ml^-1^, means the variation in recovery efficiencies observed across bacteria *spp.* using the SST approach is unlikely to compromise BSI coverage or rapid downstream analysis with a standardized AST concentration of 10^5^ c.f.u. ml^−1^ [41].

The use of horse blood offered a good surrogate to evaluate a number of different bacterial recovery methods without the need to involve human participants. Although, horse blood and human blood share a similar packed cell volume (PCV) of between 40–50 %, horse blood contains a much higher concentration range of erythrocytes than human blood, 6.0–9.6×10^12^ l^−1^ and 3.5–5.9×10^12^ l^−1^, respectively [42, 43]. As the exact erythrocyte concentration could not be quantified in the study, it is difficult to assess the impact a higher erythrocyte concentration may have had on bacterial recovery.

Another consideration is that clinically positive blood cultures are likely to contain bacteria that have undergone stress either as a result of an immune challenge or in some cases pre-exposure to antimicrobials. Clinical blood cultures may also be polymicrobial, containing more than one bacterial *spp*. and this occurs in around 11.6 % of BSI cases [44]. It is important to note that the recovery efficiencies given in this study are reflective of monomicrobial cultures only. The effect of bacterial stress and polymicrobial growth on bacterial recovery among patient blood cultures remains to be explored through future clinical studies.

Direct processing of blood culture for ID and AST is the way forward in cutting time to inform treatment decisions in bacteraemia. The recovery of bacteria from blood culture influences the quality and efficiency of the downstream analysis and therefore the recovery method must be carefully selected. Here, we have presented the efficiencies of common recovery methods, addressing a deficiency in the literature. Importantly, some ID and AST methods may perform better with different recovery methods. For instance, rapid molecular ID and AST methods would be optimal using a recovery method that enables complete removal of human cells and other PCR inhibitors. Other methods that detect ID and AST phenotypically are impacted not so much by the presence of human cells but the starting bacterial concentration. Based on the objectives of the study, the SST method was the optimal recovery method and will be further tested in combination with a rapid phenotypic detection and rapid AST system.
